# 1064 nm FT-Raman spectroscopy for investigations of plant cell walls and other biomass materials

**DOI:** 10.3389/fpls.2014.00490

**Published:** 2014-09-23

**Authors:** Umesh P. Agarwal

**Affiliations:** Fiber and Chemical Sciences Research, USDA Forest Service Forest Products LaboratoryMadison, WI, USA

**Keywords:** Raman spectroscopy, near-IR, cell walls, plants, biomass, cellulose, lignin, crystallinity

## Abstract

Raman spectroscopy with its various special techniques and methods has been applied to study plant biomass for about 30 years. Such investigations have been performed at both macro- and micro-levels. However, with the availability of the Near Infrared (NIR) (1064 nm) Fourier Transform (FT)-Raman instruments where, in most materials, successful fluorescence suppression can be achieved, the utility of the Raman investigations has increased significantly. Moreover, the development of several new capabilities such as estimation of cellulose-crystallinity, ability to analyze changes in cellulose conformation at the local and molecular level, and examination of water-cellulose interactions have made this technique essential for research in the field of plant science. The FT-Raman method has also been applied to research studies in the arenas of biofuels and nanocelluloses. Moreover, the ability to investigate plant lignins has been further refined with the availability of near-IR Raman. In this paper, we present 1064-nm FT-Raman spectroscopy methodology to investigate various compositional and structural properties of plant material. It is hoped that the described studies will motivate the research community in the plant biomass field to adapt this technique to investigate their specific research needs.

## Introduction

Raman spectroscopy is an important method for investigating various plant tissues because it provides molecular level information on composition and structure of cellular components (Atalla and Agarwal, 1985; Agarwal and Ralph, 1997, 2007; Agarwal, 2006; Gierlinger and Schwanninger, 2006; Agarwal et al., 2010, 2013a; Gierlinger et al., 2010; Schmidt et al., 2010; Hänninen et al., 2011; Sun et al., 2011; Zhanga et al., 2012). This is in contrast to techniques like light microscopy, scanning electron microscopy (SEM), transmission electron microscopy (TEM), and atomic force microscopy (AFM) which provide only morphological information of a material. Moreover, the non-destructive nature of Raman analysis along with none-to-minimal requirement of sample preparation makes it exceptionally useful for various investigations. It is well established that the information provided by Raman is complementary to information generated by infrared (IR) (Lang et al., 1986). However, in presence of water, IR has limited applicability because water-contribution is present as broad absorption bands. Additional limitation is because in an IR spectrum background variations occur with changes in refractive index of a material. In mapping applications, compared to Raman, IR has lower spatial resolution. Nevertheless, IR has been applied extensively (McCann et al., 1992; Morris et al., 2003). In the field of plant science, Raman was first applied to study tracheid cells in the xylem of woody tissues in 1980s (Atalla and Agarwal, 1985, 1986). Over the years, the field of Raman spectroscopy has continued to evolve and has come a long way since. Technological developments (Adar et al., 2007) in the fields of filters, detectors, and lasers have made Raman instrumentation more suited to investigations of plant tissue.

Although both conventional and near-IR FT-Raman spectroscopies are based on the same principle (Hendra et al., 1991), the latter differs from conventional Raman in two important ways—(1) the laser wavelength used to excite samples lies in the near-IR vs. the visible spectrum and (2) instead of using dispersive gratings, a Michelson interferometer is used to analyze scattered light to increase signal to noise ratio (Hendra et al., 1991). One of the main problems associated with the use of conventional Raman on plant materials is the very strong autofluorescence that is produced when phenolic compounds (i.e., lignin) are excited by visible light. In addition, the energies required to generate a Raman signal detectable above the autofluorescence, can cause heating and subsequent modification of the plant tissue. Near-IR excitation, particularly at 1064 nm, generates a weaker Raman signal, but more importantly, has a dramatic decrease in plant tissue autofluorescence. To be detected, this weaker Raman signal therefore requires the more sensitive interferometry scheme and subsequent FT analysis. Two of the most important advances associated with the FT approach are Jacquinot and Felgett (also known as “multiplex”) advantages (Hendra et al., 1991). The high throughput advantage of the interferometer is called the Jacquinot advantage whereas the Felgett advantage allows simultaneous detection of all the wavelengths of light. The latter is the primary reason why an FT instrument records a spectrum in a shorter time than a grating instrument. The twin advantages more than offset the loss in scattering efficiency as a result of longer wavelength excitation (compared to visible). An additional advantage of FT Raman spectroscopy is the accuracy of the wavenumber values in a spectrum. This is particularly important when spectra are to be subtracted. Previous work from our laboratory on residual lignin in kraft pulps (Agarwal et al., 2003), coated paper (Agarwal and Atalla, 1995), woody tissues (Agarwal and Ralph, 1997), photoyellowed mechanical pulps (Agarwal and McSweeny, 1997), and milled-wood lignins (Agarwal et al., 2011) demonstrated the advantage of the 1064-nm excitation Raman spectroscopy of plant materials.

In this paper, we present previous applications of 1064-nm FT Raman spectroscopy to the field of plant science as well as recent studies conducted in our laboratory. Various areas covered are (1) characteristic features of plant materials, (2) investigation of compression wood vs. normal wood (compression wood is reaction wood that is formed on the lower sides of branches and leaning trunks), (3) cellulose crystallinity, (4) lignin quantitation, (5) sampling in native (hydrated) vs. dry states, (6) chain- and local-level conformations of cellulose molecule, (7) cellulose-water interactions, and (8) low frequency region. It is hoped that the descriptions of Raman applications to these topics will demonstrate the utility of the method to the field of plant and biomass and will encourage others to apply the method.

## Materials and methods

### Materials

A large number of materials have been used to identify Raman features of various chemical constituents. These consisted of softwoods and hardwoods, milled-wood lignins, enzyme milled wood lignins, glucomannan, xylan, pectins, bleached kraft wood-pulp, WhatmanCC31 cellulose, Whatman #1 cellulose paper, sugarcane pith, and grasses. Chlorophyll and carotenoids were extracted by boiling aqueous 80% ethanol followed by boiling in CHCl_3_ (Iiyama and Wallis, 1990). Avicel was purchased from FMC corporation (Newark, DE). Most other reagents and chemicals were from Sigma-Aldrich (St. Louis, MO). Tunicate, Valonia Macrophysa, and bacterial cellulose were gifts from Prof. Akira Isogai (University of Tokyo, Japan), Dr. Noritsugu Terashima (Nagoya, Japan), and Prof. Jeffrey Catchmark (Penn State, University Park, PA), respectively. Cellulose nanocrystals (CNCs) were produced at Forest Products Laboratory using H_2_SO_4_ hydrolysis procedure (Reiner and Rudie, 2013). Cellulose II and cellulose III_I_ (cellulose II and cellulose III_I_ are crystalline forms of cellulose that differ from each other and from cellulose I which is present in nature) were generated in our laboratory by mercerization (Hirota et al., 2010) and liquid ammonia treatments, respectively. The liquid ammonia treatment was as follows. Two hundred and eighty milligram avicel was placed in a glass centrifuge tube and was slowly lowered into a dewar of liquid nitrogen. A teflon tube was attached to a lecture bottle of ammonia gas and a slow bleed of gas was introduced into the top of the tube until a block of white, frozen ammonia formed. The gas flow was stopped and most of the liquid nitrogen removed from the dewar. The sample was left to slowly warm over 4 h. As the sample warmed the ammonia block melted to a liquid which eventually began to bubble as it boiled off. Post treatment, the avicel appeared dry and very similar to its original state by the next morning. Normal wood and compression wood sections, 30 μm thick, were from black spruce and were produced using a sliding microtome. The sections were extracted first with acetone/H_2_O (9:1) and then with toluene/ethanol (2:1). The extracted sections were used for FT-Raman microprobe analysis.

### Raman experiments

Raman experiments were carried out using two instruments Bruker RFS 100 and MultiRam (Bruker Instruments Inc., Billerica, MA). Both the instruments are equipped with 1064-nm 1 W solid state Nd:YAG laser and a liquid nitrogen-cooled germanium detector. Most samples of materials in solid-state like lignin, cellulose, and milled-wood were pressed into pellets and sampled. However, where needed these samples were also sampled in water and D_2_O using shortened NMR glass tubes. Water to D_2_O exchange was carried out by first removing excess water then adding 99.9% D_2_O and subsequently stirring the sample in the sampling tube. This was followed by centrifugation (at 4000 × g) and repeating the procedure a couple of times, each time replacing the old D_2_O with fresh D_2_O. The laser power used for sample excitation was between 600 and 900 mW. Anywhere from 1024 to 16,384 scans were obtained and averaged depending upon Raman scattering of the samples and S/N ratio desired. For spatially resolved analysis (wood sections), the microscope attachment equipped with 40× objective was used in investigations. The spatial resolution of the system was ~40 μm. Areas for sampling were randomly selected based on the images of the sections. The peak height intensities were obtained by the sloping baseline method and for crystallinity measurements, as described previously (Agarwal et al., 2010).

### Scanning electron microscopy (SEM)

Scanning electron micrographs were obtained by placing X-sections of spruce wood on mounts with double-stick silver tape. Samples were gold-coated by means of a Denton Desk-1 sputter coater (Cherry Hill, NJ, USA) and examined and photographed with a Zeiss EVO 40 scanning electron microscope (Carl Zeiss SMT Inc., Thornwood, NY, USA). SEM images were obtained at several different magnifications.

## Results and discussions

1064-nm FT Raman spectroscopy is becoming increasingly useful technique in the fields of plant science and biomaterials. In the following two categories of applications are described—some that have been previously carried out and reported in literature and those that were recently completed in author's laboratory.

### Characteristic Raman features of plant materials and components

Raman spectra of plant materials are complex because they comprise of the various vibrational and rotational motions associated with many components or materials. Plant materials are composed of cellulose, hemicelluloses, lignins, extractives, pectins, water, and residual ash. Extractives are non-structural constituents that can be removed by using various solvents and consist of a wide range of organic compounds (Sjöström, 1993). These are usually removed prior to Raman analysis. To obtain useful chemical information from Raman spectra, it is necessary to find out the spectral contributions of the components of a plant material. In this paper, we have listed (Table 1) Raman characteristic bands of most of the structural components. Additionally, bands associated with chlorophyll and carotenoids are included. The band positions and spectral patterns of various components can serve as references for the interpretation of Raman spectra of biomaterials.

**Table 1:** **Raman band positions of various plant biomass components**.

**Component**	**Raman bands (cm^−1^)**	**References**
Cellulose (Whatman #1)	331 (sh), 348 (w), 381 (m), 437 (m), 459 (m), 492 (w), 520 (m), 898 (m), 971 (w), 997 (w), 1037 (sh), 1063 (sh), 1073 (sh), 1096 (s), 1121 (s), 1152 (m), 1294 (m), 1339 (m), 1380 (m), 1409 (sh), 1456 (sh), 1478 (m), 2739 (w), 2895 (vs), 2966 (sh), 3264 (w)	Agarwal and Ralph, 1997
Xylan	315 (m), 377 (w), 421 (m), 494 (s), 535 (m), 584 (w), 614 (m), 829 (vw), 900 (m), 984 (m), 1091 (s), 1126 (vs), 1217 (w), 1247 (m), 1318 (m), 1378 (m), 1413 (m), 1471 (m), 2896 (vs), 2997 (m)	Agarwal and Ralph, 1997
Glucomannan	307 (w), 346 (w), 423 (w), 492 (w), 672 (w), 897 (w), 1089 (m), 1121 (m), 1267 (m), 1374 (m), 1463 (m), 2918 (vs), 2935 (sh)	Agarwal and Ralph, 1997
Lignin (softwood)	361 (w), 384 (w), 457 (vw), 491 (vw), 534 (vw), 557 (vw), 588 (vw), 637 (vw), 731 (w), 787 (w), 811 (sh), 895 (vw), 928 (vw), 975 (vw), 1033 (w), 1089 (w), 1136 (m), 1192 (w), 1226 (vw), 1272 (m), 1298 (sh), 1334 (m), 1363 (sh), 1392 (sh), 1430 (w), 1453 (m), 1508 (vw), 1597 (vs), 1621 (sh), 1662 (s), 2845 (m), 2890 (sh), 2940 (m), 3008 (sh), 3071 (m)	Agarwal et al., 2011
Lignin (hardwood)	369 (m), 417 (vw), 431 (vw), 447 (vw), 461 (vw), 472 (vw), 490 (vw), 503 (vw), 522 (sh), 531 (m), 588 (w), 597 (m), 638 (w), 727 (w), 797 (w), 899 (w), 918 (sh), 984 (sh), 1037 (m), 1088 (w), 1130 (m), 1156 (sh), 1190 (w), 1224 (w), 1272 (m), 1331 (s), 1367 (sh), 1395 (sh), 1426 (w), 1455 (m), 1501 (vw), 1595 (vs), 1620 (sh), 1661 (s), 2847 (m), 2893 (sh), 2939 (m), 3003 (sh), 3068 (m)	Agarwal et al., 2011
Lignin (sugarcane pith)	370 (m), 416 (vw), 529 (sh), 545 (sh), 590 (sh), 603 (sh), 713 (sh), 835 (sh), 863 (m), 919 (w), 982 (w), 1039 (w), 1171 (s), 1203 (m), 1266 (m), 1378 (w), 1453 (m), 1517 (w), 1589 (sh), 1605 (vs), 1631 (s), 1697 (m), 2835 (w), 2933 (m), 2970 (m), 3017 (sh), 3067 (w)	Author's unpublished work
Pectins	340, 372, 441, 486, 537, 621, 686, 710, 750 (sh), 775, 795, 834, 853, 887, 953, 990, 1030, 1050, 1079, 1105, 1145, 1254, 1330, 1393, 1740, 2941	Čopíková et al., 2003
Pigments^a^ (grass; chlorophyll and carotenoids)	319 (w), 490 (vw), 618 (w), 706 (vw), 796 (w), 981 (w), 1003 (w), 1051 (w), 1090 (sh), 1131 (sh), 1158 (vs), 1134 (w), 1158 (s), 1190 (m), 1201 (sh), 1248 (m), 1270 (m), 1295 (w), 1356 (w), 1373 (m), 1388 (w), 1452 (m), 1527 (s), 1604 (s), 1630 (sh), 2852 (sh), 2880 (sh), 2907 (m), 2932 (m)	Caia et al., 2002 Author's unpublished work

a *Due to underlying fluorescence not all bands could be detected*.

### Investigations of compression wood vs. normal wood

**Figure 1:**
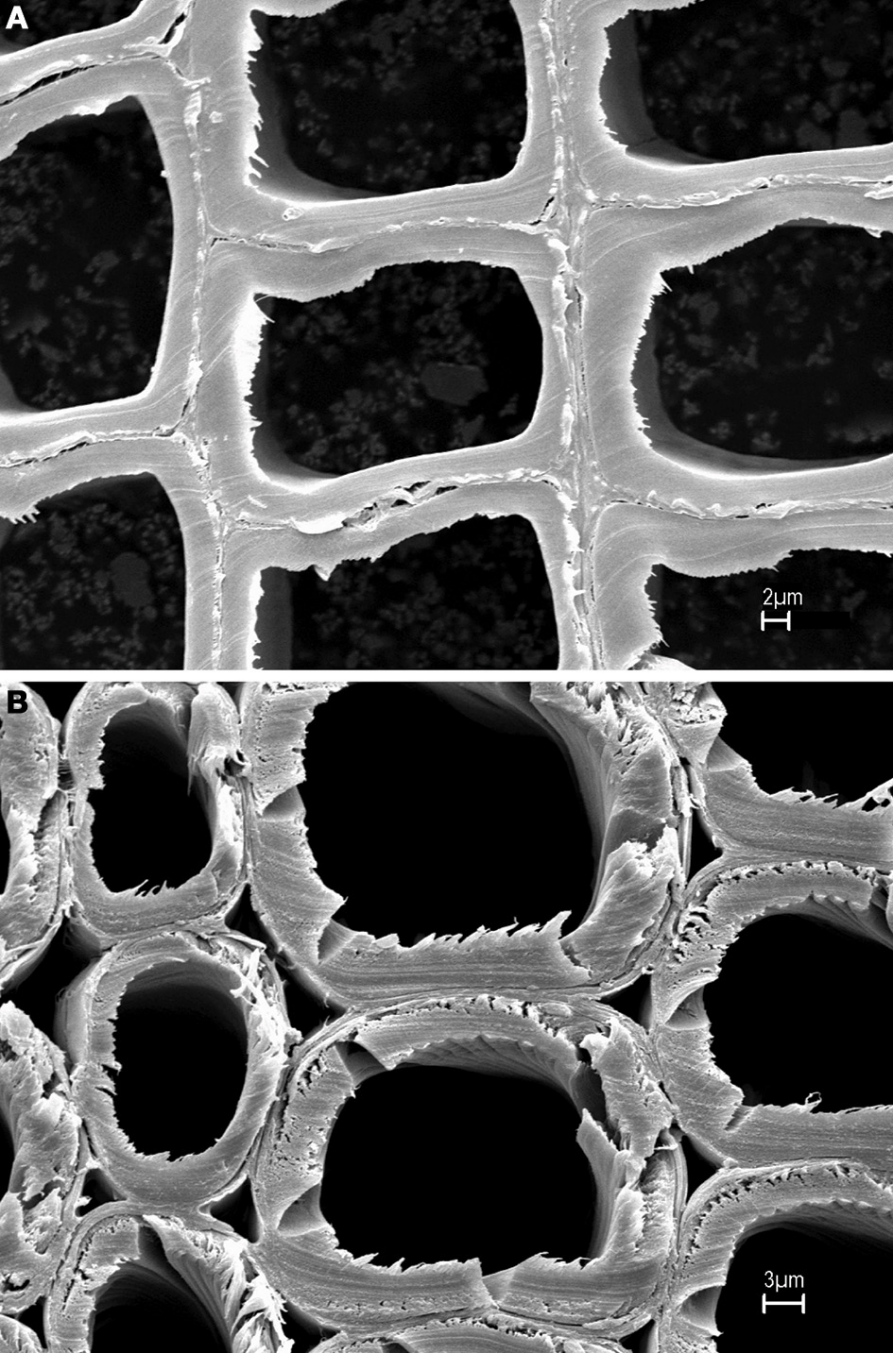
**SEMs of normal (A) and compression wood (B)**.

**Figure 2:**
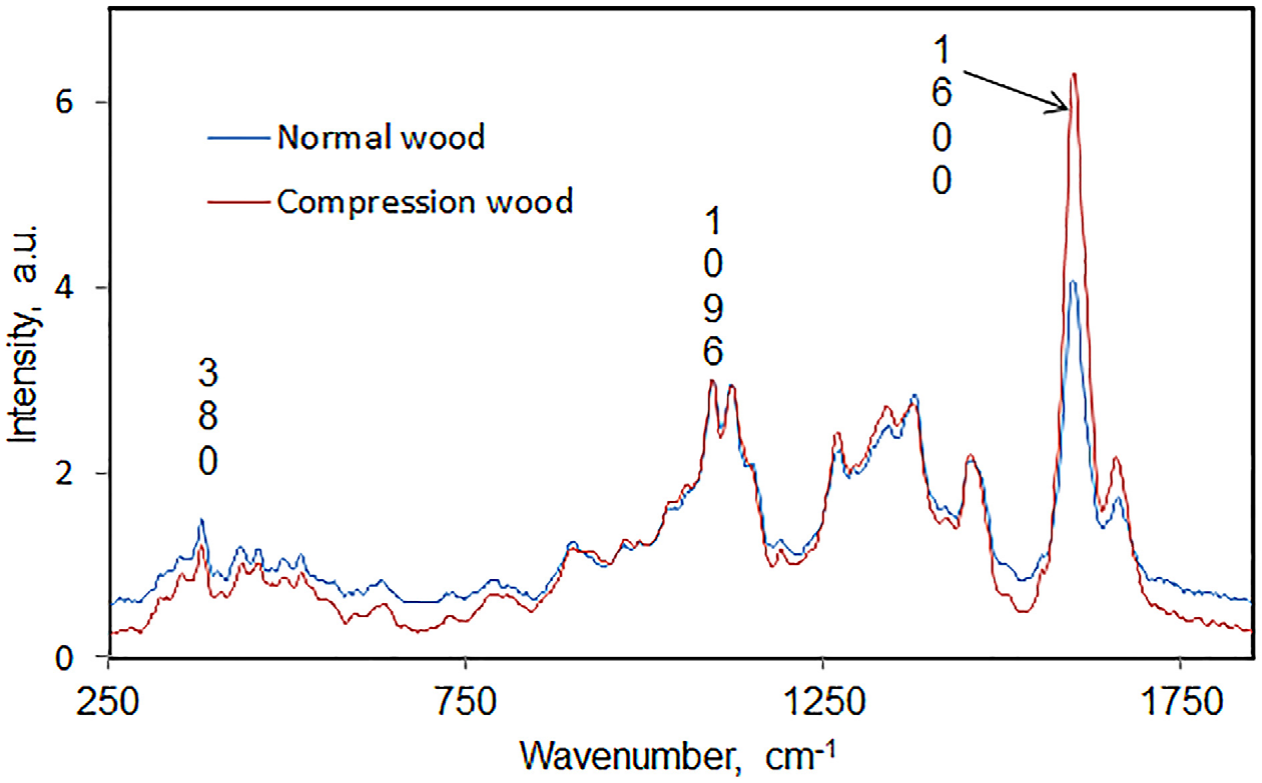
**Average Raman spectra, in the region 250–1850 cm^−1^, comparing normal and compression wood of black spruce**.

In author's laboratory, an FT-Raman microprobe (Raman instrument with an attached optical microscope) was used to investigate differences between early wood and late wood cells of black spruce. The conclusion was that the two had similar chemical composition and crystallinity. Considering that compression black spruce wood contains more lignin and less cellulose (Timell, 1982) compression-wood and normal-wood cells were analyzed for lignin-to-cellulose ratio and cellulose crystallinity. Solvent extracted air-dried 30 microns thick cross sections of normal and compression woods (SEMs in Figure 1) were directly analyzed using a 40× microscope objective and spectra were obtained from several regions of the sections. Lignin-to-cellulose ratio (Table 2) was calculated by taking the ratio of the peak heights at 1600 and 1096 cm^−1^, which represented lignin and cellulose, respectively, (Table 1; Agarwal and Ralph, 1997). Although this ratio varied significantly among the sampled-locations in compression tissue, compared to normal-wood, on average, the ratio was found to be two times higher (Table 2, Figure 2). This is in agreement with previous finding that compression wood contains less cellulose and more lignin (Timell, 1982). However, there was no difference between the cellulose crystallinity (as measured by the 380/1096 peak ratio Raman method, *vide infra*) of compression-wood and normal-wood. The conclusion on crystallinity is also supported by earlier findings (Newman, 2004; Agarwal et al., 2013a).

**Table 2:** **Lignin-to-cellulose ratio in normal and compression woods**.

**Sample type**	**Ratio 1600/1096**
**NORMAL WOOD**	
Area 1	1.48
Area 2	1.51
Area 3	1.64
Area 4	1.69
Average	1.58 ± 0.1
**COMPRESSION WOOD**	
Area 1	3.28
Area 2	3.21
Area 3	3.37
Area 4	3.32
Area 5	3.42
Area 6	3.05
Area 7	3.31
Area 8	3.52
Area 9	3.32
Area 10	2.67
Area 11	2.13
Area 12	2.40
Area 13	3.10
Area 14	3.38
Area 15	2.83
Average	3.09 ± 0.4

### Cellulose crystallinity

In 2010, a new method based on 1064-nm FT-Raman was published (Agarwal et al., 2010) for estimating cellulose crystallinity. The method developed in author's laboratory, is based on the following equation where crystallinity was found to be directly related the Raman peak-height ratio (I_380_/I_1096_).

$${\text{Cr}}_{\text{Raman}}=[({\text{I}}_{380}/{\text{I}}_{1096})-0.0286]/0.0065$$

**Figure 3:**
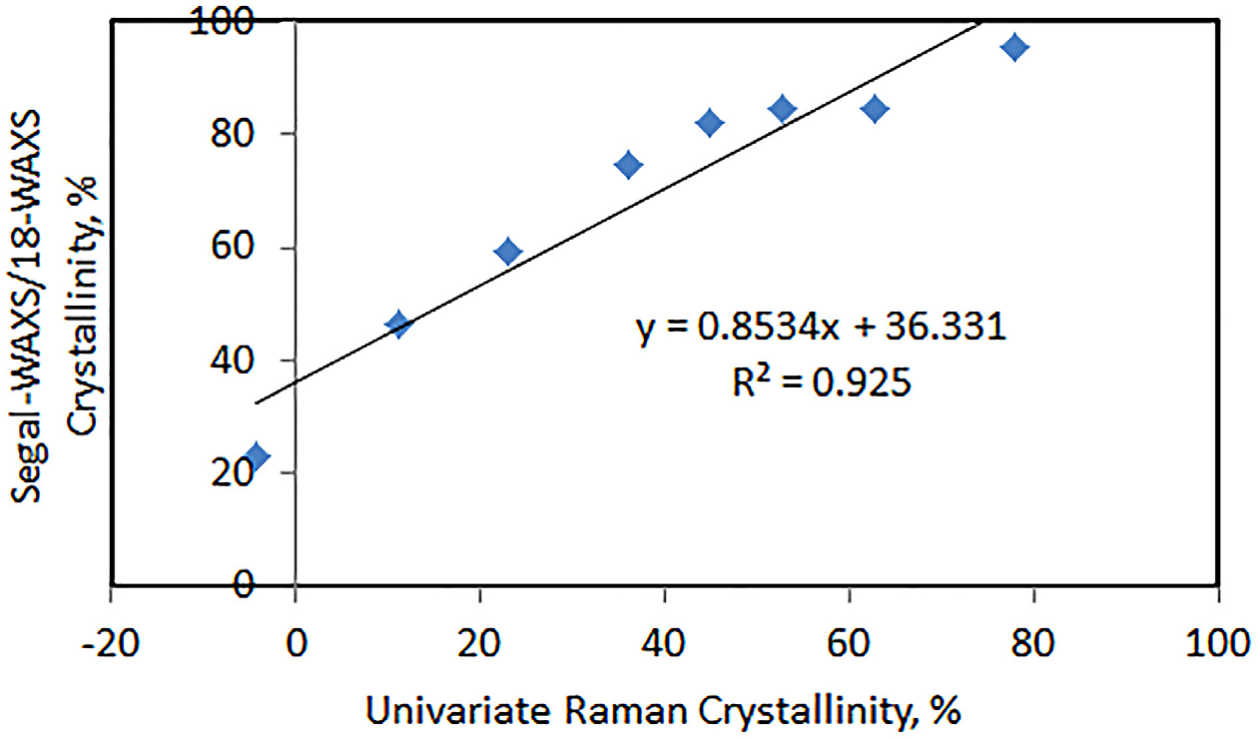
**Correlation between 18-Segal-WAXS and univariate Raman crystallinities**.

The estimation of cellulose crystallinity by Raman can be carried out for cellulose products and cellulose composites. This method has been successfully applied in our laboratory to calculate crystallinity in cellulose and plant materials (Agarwal et al., 2013a,b,c; Qing et al., 2013) as well as in other laboratories (Zhu et al., 2011; Barnette et al., 2012; Thygesen and Gierlinger, 2013). The Raman method was compared to the Segal-WAXS method and the two methods were found to be well correlated (Figure 3; Agarwal et al., 2013a). However, it is important to note that the absolute values of crystallinities between the two methods are different due to the fact that initially when the Raman method was developed (Agarwal et al., 2010) it was correlated with the 21-Segal-WAXS not 18-Segal-WAXS. In 21-Segal-WAXS, 21 represents the 2θ position where the amorphous contribution was measured and subtracted from the peak height. Obviously, because larger diffraction intensity was subtracted compared to the value at 18° (2θ), 21-Segal-WAXS crystallinity values (and therefore Raman estimations) were significantly lower compared to 18-Segal-WAXS.

Some of the major advantages of the Raman method are that it works even when water is present and no additional correction is needed for the presence of amorphous cellulose or guaiacyl-lignin (coniferous or gymnosperms). Nevertheless, a small correction is required when a sample contains syringyl-lignin (angiosperms and herbaceous plants) and hemicelluloses. The method is, however, restricted for materials whose spectra contain significant level of fluorescence (usually colored samples). This situation can be remedied by either bleaching or removing lignin (usually the source of fluorescence).

**Figure 4:**
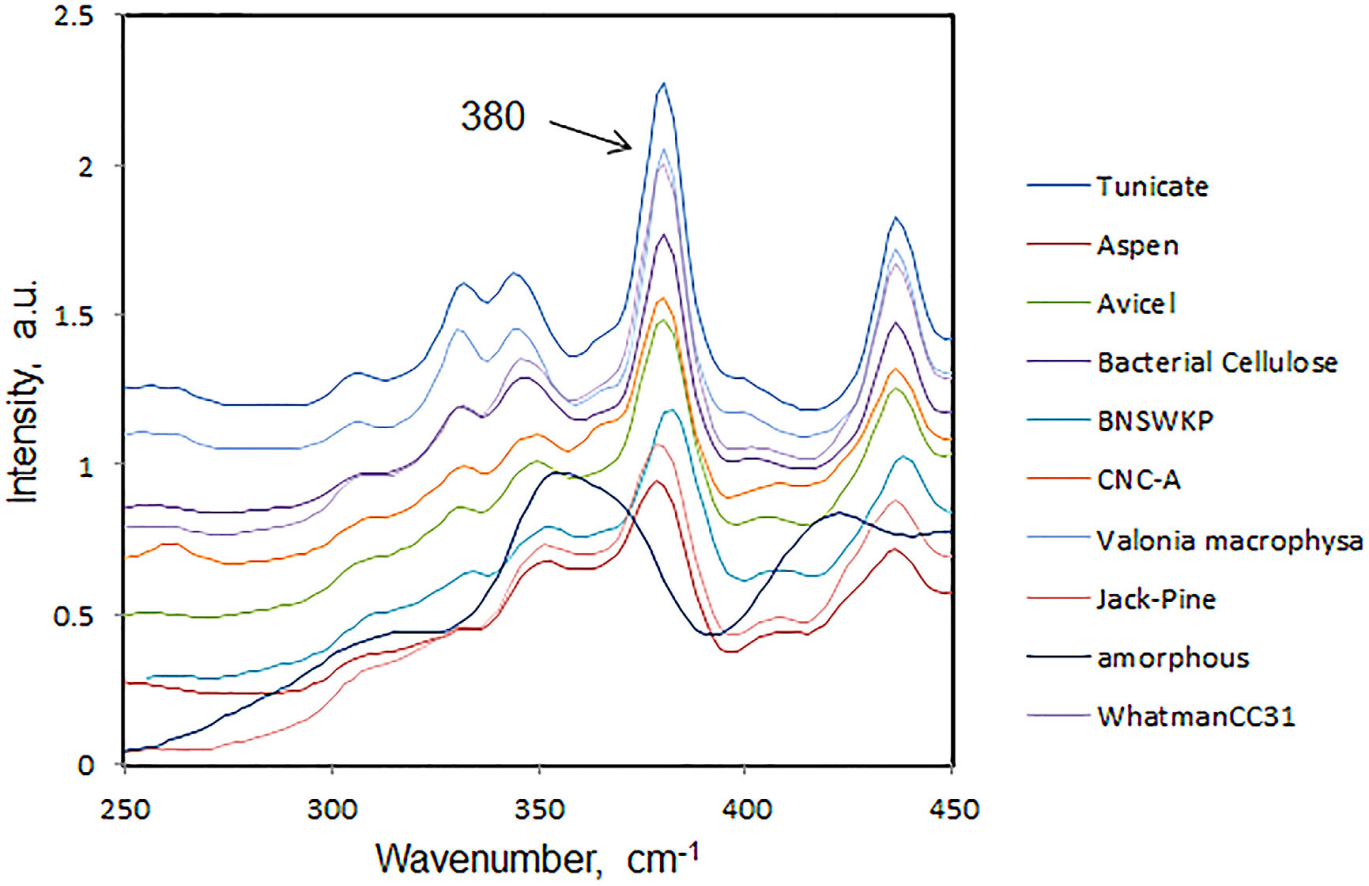
**380 cm^−1^ region of Raman spectra of a number of cellulose samples**.

In Figure 4, Raman spectra from a number of cellulose and plant materials are shown in the region 250–450 cm^−1^. The spectra have been normalized on the 1096 cm^−1^ band which in cellulose molecule has been assigned to C-O and C-C bond stretches (Wiley and Atalla, 1987). In Figure 4, the 380 cm^−1^ band intensity order represents the order of crystallinity. The declining order of crystallinity is as follows—tunicin cellulose (marine animal) > valonia macrophysa (algae) > bacterial cellulose > WhatmanCC31 > CNC (wood pulp) > avicel > softwood kraft pulp > wood cellulose > amorphous cellulose.

### Lignin quantitation

**Figure 5:**
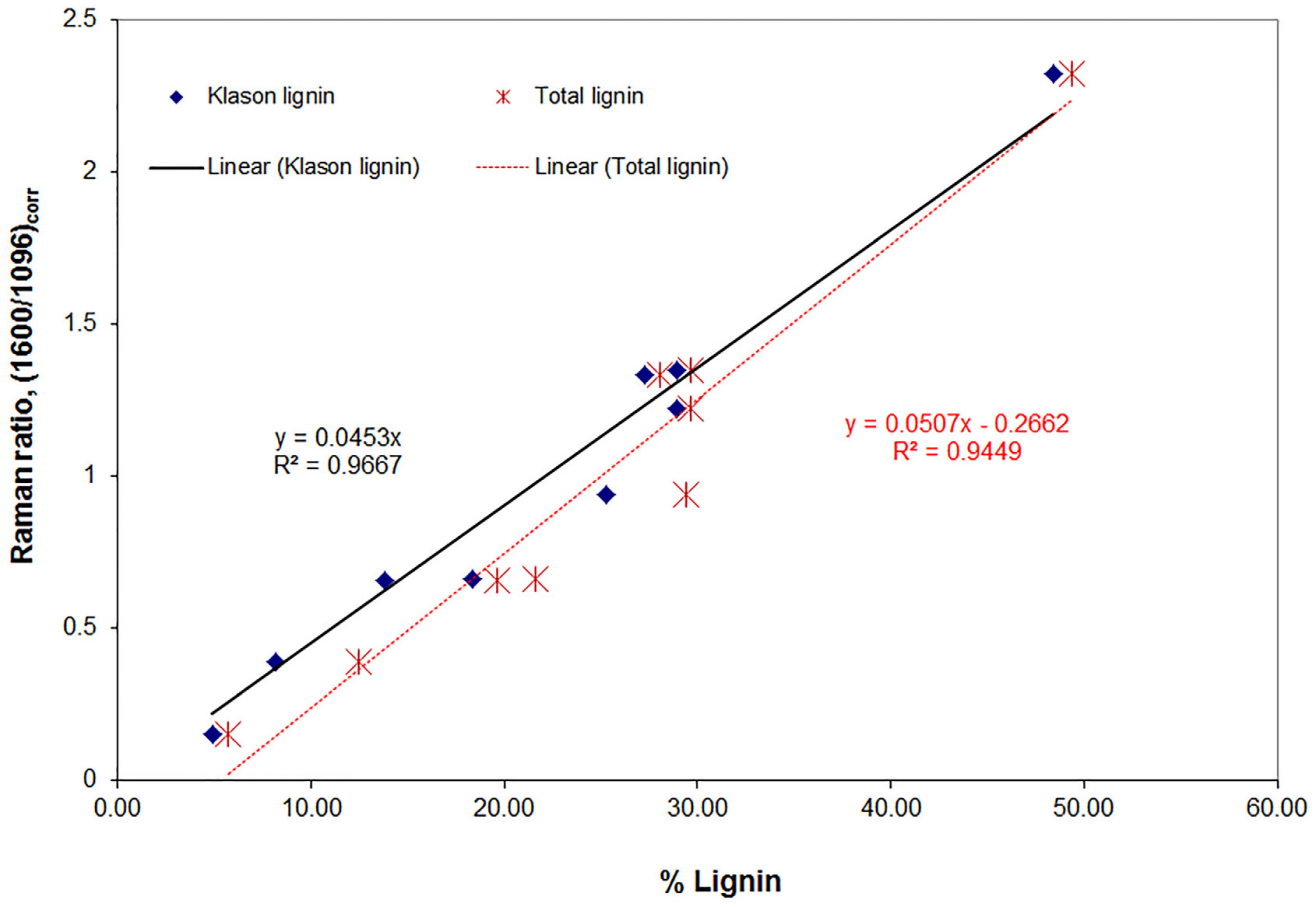
**Correlations of lignin's 1600 cm^−1^ band intensity with Klason and total lignin for various lignocellulosic materials**. Klason lignin (%) is listed in parentheses. Black spruce MWEL (48.4), 2 samples of southern pine (28.9), black spruce (27.3), white oak (25.2), aspen (18.3), corn stalk (13.8), partially delignified black spruce (8.1), and unbleached kraft pulp (4.9).

In light of structural complexity of biomass, quantitation of lignin in a variety of wood and plant cell walls is difficult. A novel approach that successfully quantified lignin was developed and based upon the lignin band intensity at 1600 cm^−1^. However, chromophores and lignin structures conjugated to aromatic ring are over represented in the band intensity and need to be reduced in order to quantify lignin from Raman spectra. Their contributions to the 1600 cm^−1^ lignin band were minimized using a number of chemical treatments. Lignin structures containing conjugation to aromatic rings are removed/reduced with alkaline hydrogen peroxide (H_2_O_2_), diimide (N_2_H_2_), and sodium borohydride (NaBH_4_) treatments. Using a variety of material-samples with varying range of lignin composition (4.9–48.4% composition), a good linear correlation between Raman and Klason or total lignins was developed (Figure 5; coefficients of determination *R*^2^ 0.97 and 0.95, respectively) (Agarwal, 2011).

### Sampling in native (hydrated) vs. dry states

One of the strength of Raman spectroscopy is the ability to analyze a sample in its native state. This means that water as well as other components that are not of interest need not be removed from the sample. The only spectral requirement is that the sample-band of interest must not overlap with the Raman contribution of some other sample component. In native state, all plant biopolymers exist in the fully hydrated form. When isolated and dried their original state of aggregation is usually altered and consequently, some information is lost. Therefore, to investigate the native state it's imperative that the materials be analyzed in the never-dried state and preferably, in presence of other components.

**Figure 6:**
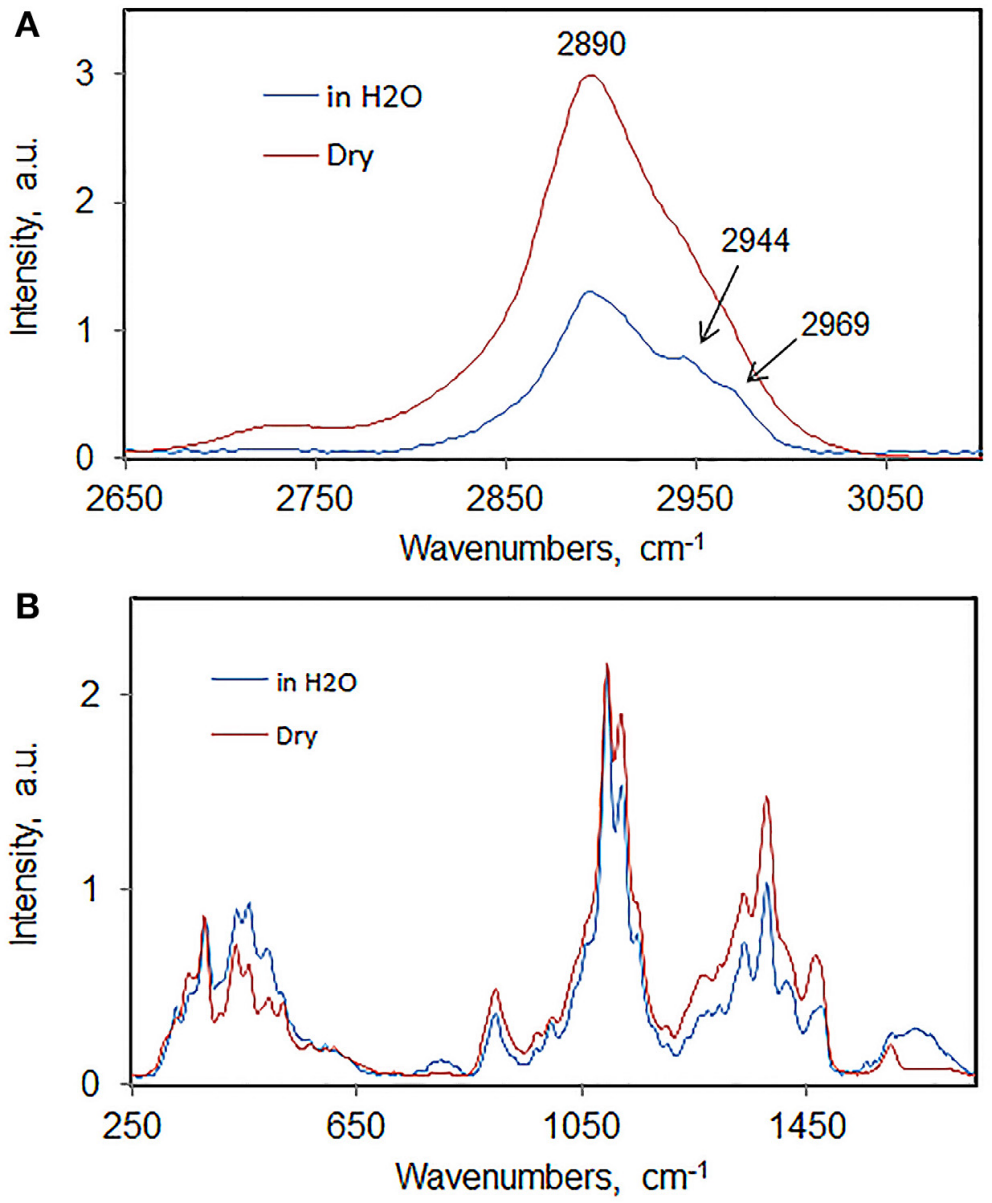
**Spectra sets of never-dried and dried aspen woodcellulose; (A) 2650–3100 cm^−1^, (B) 250–1550 cm^−1^**.

As an example, Raman spectra of wood-cellulose in never-dried and dried states are compared in Figure 6. The spectra are overlaid (after normalization to the 1096 cm^−1^ band) so that small differences can be noted. In the spectra of native vs. dried state a number of small changes were noted. In the C-H stretch region (Figure 6A), the prominent shoulders present in hydrated state at 2944 and 2969 cm^−1^ were detected. These can be barely seen when the sample is air-dried at room temperature. This spectral modification is likely to result from the change in the conformation of cellulose molecules in dry state. The intensity changes suggest that the chains have higher degree of order in the wet state. The latter observation was further supported by the comparison of the spectral data in the region 250–1750 cm^−1^ (Figure 6B). Here, in the dried state, peaks at 330, 1048, 1096, 1123, 1158, and 1430 cm^−1^ become less resolved. On the contrary, the features at 357 and 900 cm^−1^ became more intense upon drying. The “increase-in-intensity” behavior of the bands seems to be associated with increased-disorder in cellulose. From such structural information, it's clear that the FT-Raman is capable of distinguishing small molecular level changes which may not be discernable using other techniques.

### Chain- and local-level conformations

**Figure 7:**
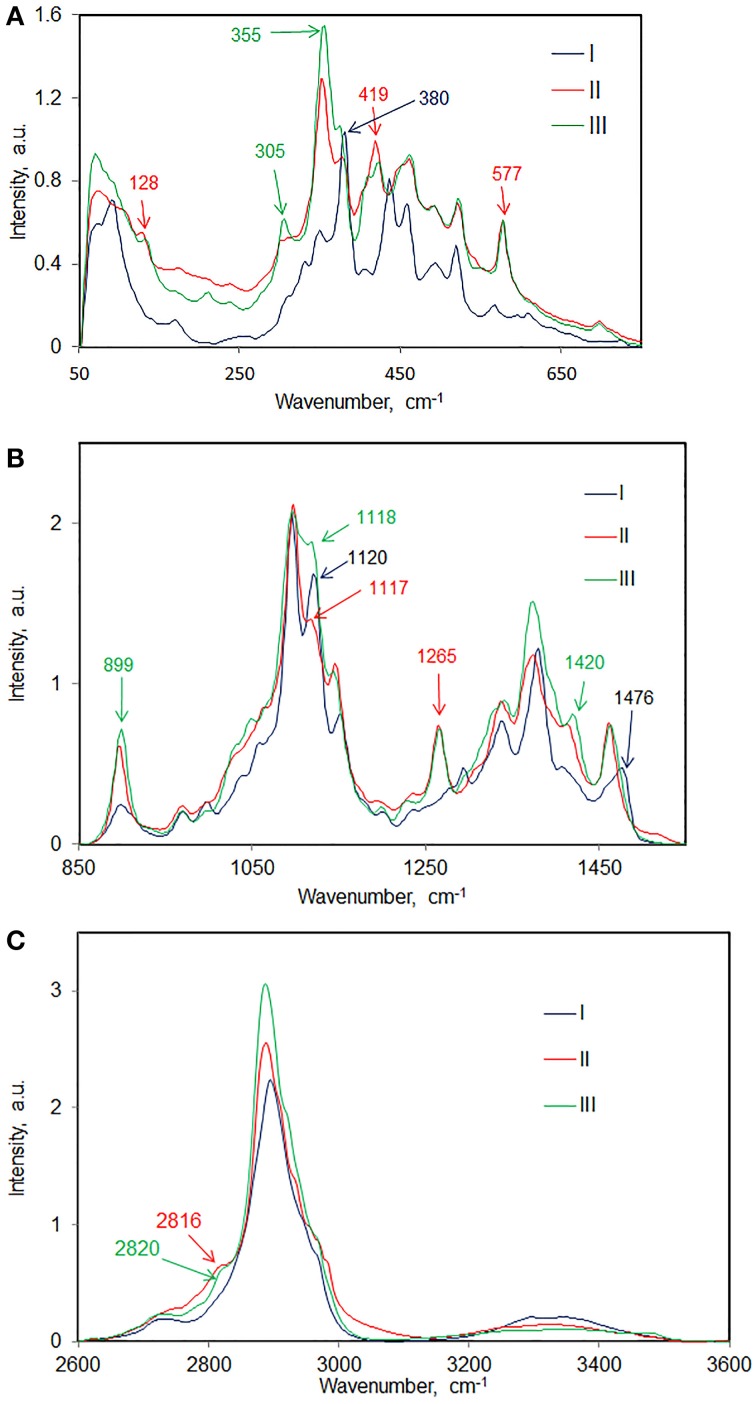
**Cellulose I, cellulose II, and cellulose III_I_ compared in various spectral regions; (A) 50–750 cm^−1^, (B) 850–1550 cm^−1^, (C) 2600–3600 cm^−1^**.

Raman spectroscopy is well suited to study different chain conformations in polymers (Hahn et al., 2001). In the area of celluloses, it is well established that different chain conformations are present in cellulose polymorphs cellulose I and cellulose II (Atalla et al., 1980). Spectral changes reflecting such conformational differences between cellulose I and cellulose II have been reported (Atalla and Dimick, 1975). In Figure 7 some of the most prominent spectral differences representing molecular conformational variations between cellulose I, cellulose II, and cellulose III_I_ are annotated. More detailed information can be noted from Table 3 where peak positions and relative intensities in the spectrum of each avicel cellulose polymorph are listed. In some spectral regions the differences are more prominent than in others. Comparison of such data and spectral evaluation suggested that in each case the cellulose spectral information between cellulose II and cellulose III_I_ polymorphs is similar and that in each case the spectral information differs from that of cellulose I. However, the important question that still remains to be answered is the manner in which the chain-conformation in cellulose II differs from that in cellulose III_I_.

**Table 3:** **Comparing Raman spectral data for avicel polymorphs (I, II, and III_I_), cm^−1^**.

**Avicel (I)**	**Avicel (II)**	**Avicel (III_I_)**	**Avicel (I)**	**Avicel (II)**	**Avicel (III_I_)**
–	3490 (sh)	3482 (sh)	1236 (w)	1236 (w)	1229 (w)
–	3446 (w)	–	1201 (w)	1193 (w)	1200 (w)
3374 (sh^*^)	3329 (w)	–	1151 (m)	1146 (m)	1144 (m)
–	–	–	1120 (s)	1117 (sh)	1118 (sh)
3350 (w)	–	3353 (w)	1096 (vs)	1096 (vs)	1096 (vs)
3295 (vw)	–	–	1058 (sh)	1058 (sh)	–
3264 (vw)	3263 (vw)	3257 (vw)	–	–	1048 (sh)
–	2984 (sh)	–	997 (w)	–	–
2969 (sh)	2970 (sh)	2968 (sh)	970 (w)	969 (w)	969 (w)
–	–	–	913 (sh)	–	
–	2956 (sh)	–	898 (m)	897 (m)	899 (m)
2947 (sh)	–	2940 (sh)	724 (vw)	–	
–	–	–	–	697 (w)	698 (w)
–	2935 (sh)	–	609 (vw)	–	–
–	–	–	595 (vw)	–	–
–	–	2922 (sh)	–	577 (m)	578 (m)
–	2911 (sh)	–	567 (w)	–	–
2894 (vs)	2888 (vs)	2887 (vs)	–	–	550 (w)
2871 (sh)	–	–	520 (m)	522 (m)	522 (m)
–	–	–	493 (w-m)	491 (w)	–
–	2816 (sh)	2820 (sh)	458 (m)	461 (m)	462 (m)
–	2776 (sh)	2781 (sh)	436 (m-s)	–	436 (m-s)
–	2744 (sh)	–	–	419 (m)	423 (m)
2734 (sh)	–	–	406 (vw)	–	–
2716 (w)	2720 (w)	2725 (w)	380 (s)	377 (m-s)	374 (m-s)
1476 (m)	–	–	350 (m)	353 (s)	355 (s)
1460 (sh)	1462 (m-s)	1464 (m-s)	331 (w)	299 (sh)	305 (m)
–	–	1420 (sh)	258 (w)	–	–
–	–	–	–	238 (w-m)	238 (w-m)
–	–	–	–	210 (w)	210 (w)
1408 (sh)	1413 (sh)	1408 (sh)	170 (w-m)	173 (w)	173 (w)
1380 (m-s)	1374 (m-s)	1373 (m-s)	154 (vw)	–	–
1338 (m)	1337 (m)	–	140 (vw)	128 (m)	133 (m)
1294 (w)	–	–	92 (m)	92 (sh)	105 (sh)
–	1265 (m)	1266 (m)	–	–	–

* *Relative band intensity; vs, very strong; s, strong; m, medium; w, weak; vw, very weak; sh, shoulder*.

Further information on structural differences can be obtained from the O-H stretch region. However, in the 1064 nm FT-Raman, the sensitivity of the detector is low in this region which is the reason why the cellulose O-H bands have low intensity. Nevertheless, differences between sample spectra can be still noted. In Table 3, spectra of avicel II and avicel III_I_ contain O-H stretch bands at 3490 and 3482 cm^−1^, respectively. Absence of 3480 cm^−1^ feature in the spectrum of avicel I indicated differences in H-bonding. Differences in the lower wavenumber O-H Raman bands existed as well (Table 3). In the C-H stretch region, none of the polymorphs have all the vibrational modes occurring at similar positions (Table 3, Figure 7C) although some of the C-H stretch modes have similar positions (e.g., shoulder at ~2970 cm^−1^). The existence of the frequency differences in the C-H region is indicative of existence of different chain conformations in polymorphs. C-H vibrations are known to be highly sensitive to the conformation of the chain (Snyder et al., 1982) and in simple carbohydrates, the CH_2_ group configuration with respect to the anhydroglucose unit is likely to be important.

**Figure 8:**
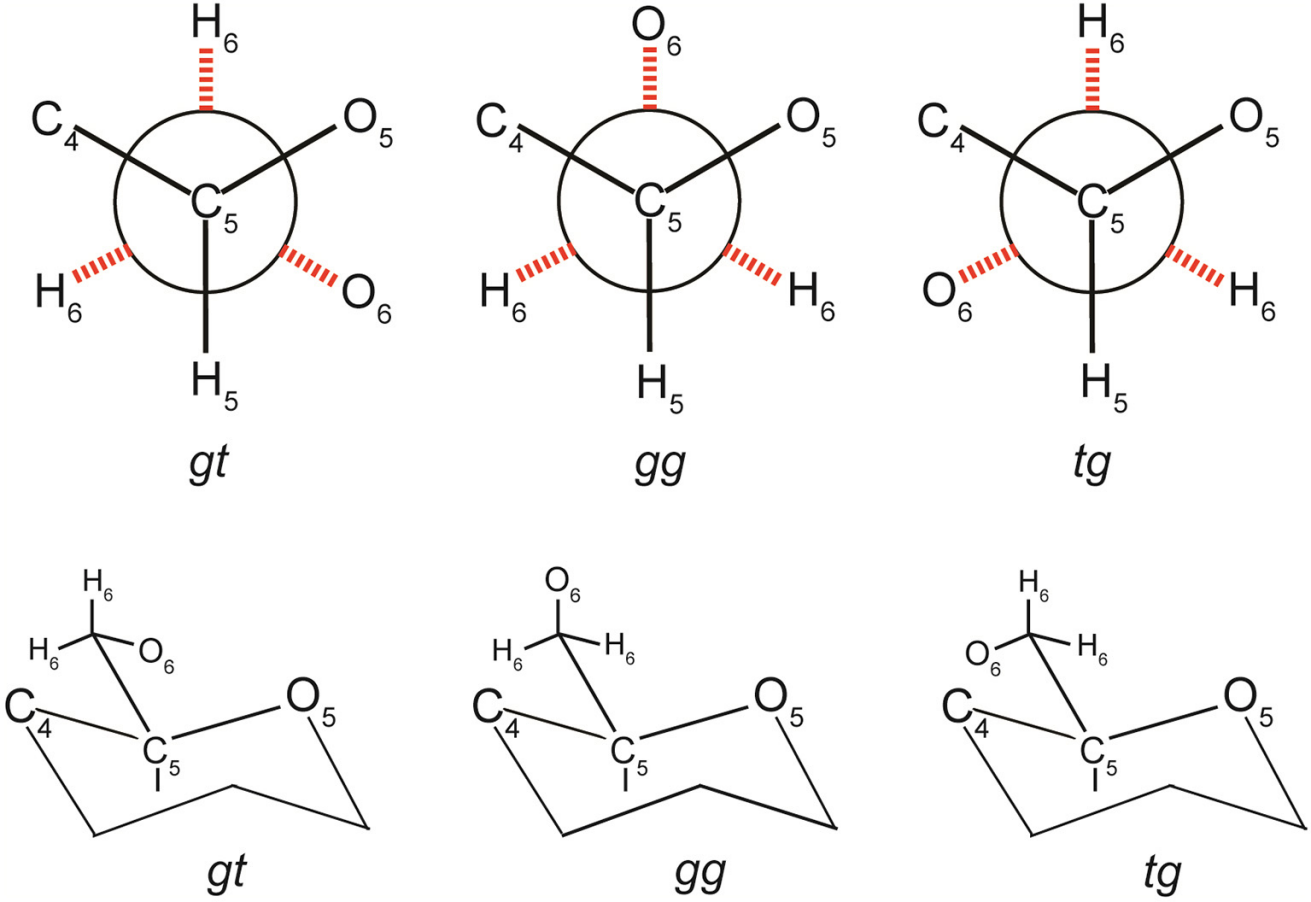
**Conformations of CH_2_OH group—*gt, gg*, and *tg***.

In the 1400–1500 cm^−1^ region, where the dominating contribution is from CH_2_ scissor mode, avicel I shows two bands whereas only one band is seen for the other two polymorphs (Table 3, Figure 7B). The scissor mode bands at 1476 and 1461 cm^−1^ have been assigned to *tg* and *gg* conformers of the CH_2_OH group (Figure 8; Agarwal and Ralph, 2014). This indicated that in avicel I cellulose structure has both *tg* and *gg* CH_2_OH conformers whereas avicel II and avicel III have only the *gg* conformer. In the rest of the Raman region below 1400 cm^−1^, there are at least four sets of bands that are only detected in cellulose avicel II and avicel III_I_ and not in avicel I. These are located at ~1265, ~697, ~577, and ~420 cm^−1^. Moreover, there are bands whose frequencies are similar to that of avicel I but the band-profiles are different (e.g., 1120 cm^−1^, Figure 7B).

The aforementioned seems to suggest that avicel II and avicel III_I_ structures may be similar because the band positions and profiles of a number of bands are similar. However, further analysis of the Raman spectra suggests that this is not the case. Although similarities exist, so do differences. For instance, compared to avicel III_I_, the spectrum of avicel II does not have bands at 3353, 2922, 1420, 1048, 550, 436, and 105 cm^−1^. Analogously, spectral features of avicel II that are missing from the spectrum of avicel III are follows—3446, 3329, 2984, 2956, 2935, 2911, 2744, 1337, and 491 cm^−1^. The situation is further complicated by the fact that all the polymorphs of avicel are expected to contain both the crystalline and the amorphous forms. The latter phase when present, will contribute and broaden the spectral features.

However, in naturally occurring celluloses which are all believed to be cellulose I, the question of the chains having variable conformations has arisen from author's recent work. Such variability is expected to be cellulose-source dependent and likely to be due to the fact that cellulose is synthesized in the context of biological function (plant/species function and reaction to external stimuli). Various cellulose molecules having one dimensional order (along the chain), present in the hydrated form, can be aligned to each other laterally, and be aggregated. This is the native cellulose structure and differs from the cellulose I structure because chain conformation and the lateral order (degree of order or crystallinity) depend upon the source of natural cellulose. It is envisioned that in such aggregates the multiple conformations of cellulose molecules are stabilized with the help of H-bonds between water-cellulose and cellulose-cellulose molecules. As the author discussed above, there are several bands in the spectra that are sensitive to overall chain conformation. On the other hand, bands that are sensitive to local conformation (bond-level or anhydroglucose unit-level) also existed. For instance, we recently discovered (Agarwal and Ralph, 2014) that the band position of the −CH_2_ scissor mode is sensitive to conformation of −C(6)H_2_OH (local conformation) with respect to the glucose unit. The conformations *gg, gt*, and *tg* (Figure 8) generated −CH_2_ scissor-mode vibrations at 1460, 1470, and 1480 cm^−1^, respectively. The conformation-variability detected in Raman has important implications for cellulose ultrastructure and reactivity because the—CH_2_OH group is involved not only in intra- and inter-molecular H-bonding but also is the principal site of chemical and biological modifications when cellulose is used industrially.

### Water removal by D_2_o

**Figure 9:**
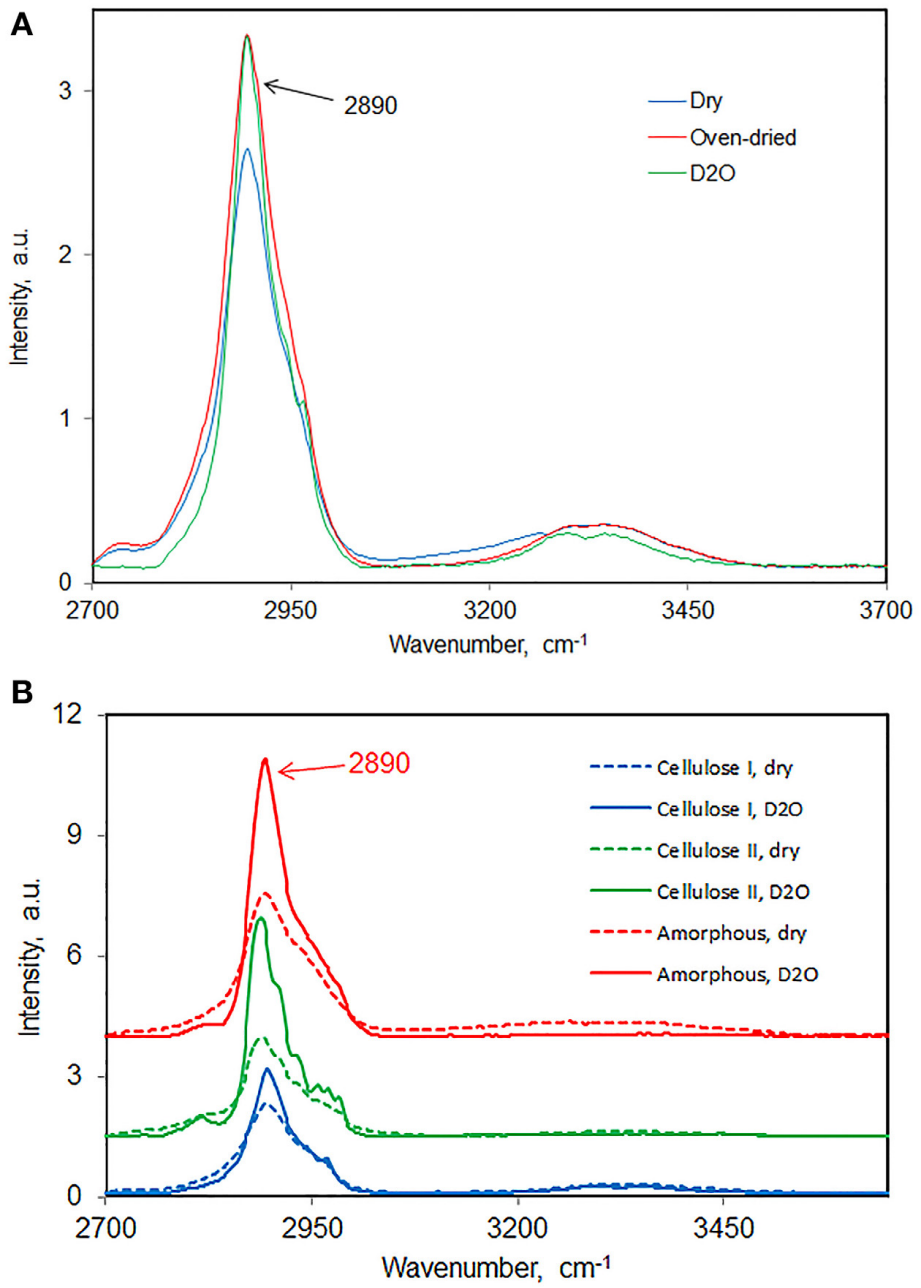
**Effect of water removal on 2890 cm^−1^ band intensity**. **(A)** BNSWKP, **(B)** cellulose I, cellulose II, and amorphous cellulose.

There are three different kind of water in cellulose and cellulose materials—free water, freezing bound-water, and non-freezing bound-water (Nakamura et al., 1981). Given the importance of water's influence on cellulose structure, it's important to know accurately how much water is present under a given set of conditions. Although traditional moisture measurements can be used but they have limitations in the sense that water may or may not be completely removed depending upon among other things the cellulose crystallinity of a material. It seems FT-Raman methodology has the potential to be developed to determine the amount of water that is present in a sample. Here, results are presented that show that when the bleached northern-wood soft-wood kraft pulp (BNSWKP) was (a) heated in an oven for long duration or (b) immersed in D_2_O, both methods removed water equally well from pulp (Figure 9A). In Figure 9A where the Raman spectra in the region 2600 and 3700 cm^−1^ are plotted, the intensity increase in the C-H stretch region was similar between oven-dried and D_2_O-immersion methods. Changes in 2890 cm^−1^ band intensity as a function of variation in moisture content are based on the phenomenon of “self-absorption” (Agarwal and Kawai, 2005). The latter has to do with the absorption of Raman scattered photons, in near-IR, by water molecules. The intensity-increase in the C-H stretch region is directly proportional to the amount of water removed; larger amount of water removal leads to larger increase in the C-H band intensity.

For some other celluloses, increase in 2890 cm^−1^ peak intensity is reported in Figure 9B—cellulose I (avicel), cellulose II (avicel), and amorphous cellulose. Compared to avicel (cellulose I), Cellulose II, and amorphous cellulose adsorb more water. The latter may have to do with existence of less accessible (to water) structure in avicel which is significantly crystalline. Further, if one considers that water diffuses only into the non-crystalline regions, it would imply that compared to cellulose I cellulose II contains significantly larger number of regions that are non-crystalline.

### Cellulose-water interactions: effect of wet-dry cycles

In pulp and paper field, study of cellulose-water interactions is an important area of research because dried cellulose fibers make weak paper (phenomenon of hornification). In the past, studies of cellulose water interactions have proven difficult due to complexity of this topic and lack of techniques to provide useful information. 1064-nm based Raman seems to be an advance because cellulose-water interactions induced changes that can be detected in the spectra. Such changes are largely present as changes in band profiles (contours and shapes). Investigation of the latter became feasible due to better S/N ratio of the spectral features in FT-Raman spectroscopy. However, extent to which such changes can be accurately interpreted in terms of molecular information remains to be seen, but this approach seems to hold significant promise.

**Figure 10:**
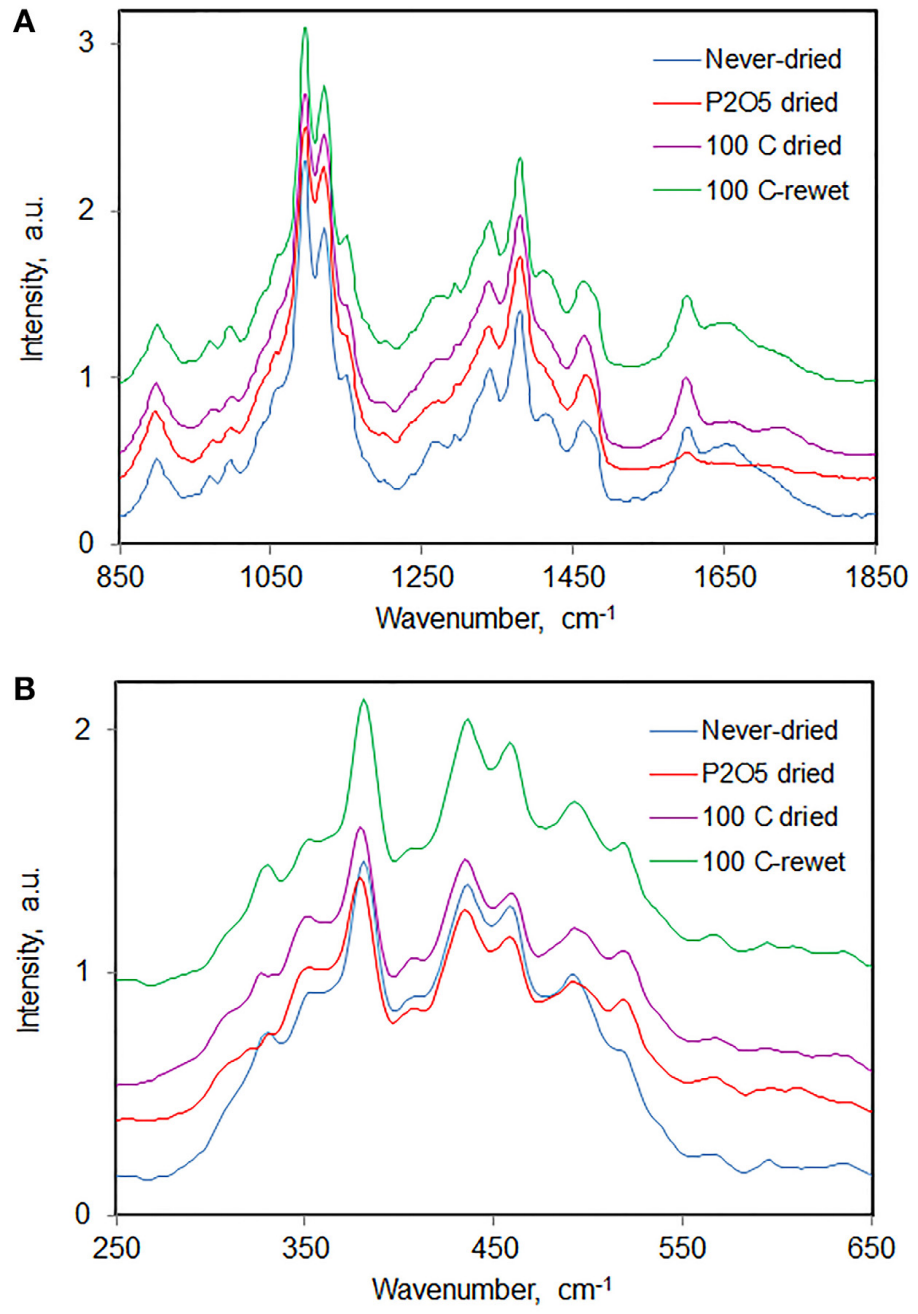
**Raman spectra of Jack pine holocellulose in never-dried, dried, and dried-then-rewet states; (A) 850–1850 cm^−1^, (B) 250–650 cm^−1^**.

As an example we report here the observations from a study that was carried out to find out what happens spectrally when one carries out drying and rewetting cycles on a sample of Jack pine holocellulose pulp from which most hemicelluloses have been removed (called wood-cellulose). In the Raman spectrum of Jack pine wood (Figure 10) almost all the observed peaks belonged to cellulose. Upon drying never-dried cellulose, either over P_2_O_5_ or at 100°C, the never-dried sample spectrum became less resolved overall (Figure 10), 350 cm^−1^ band became more intense, and bands at 1478, 1275, and 381 cm^−1^ shifted to lower frequencies. On the contrary, band at 970 cm^−1^ shifted to higher frequencies. The spectral changes were mostly reversed when the dried wood-cellulose was rewet (Figure 10), and the changes were reproducible upon an additional repeated cycle of drying and rewetting. The lower spectral resolution indicated that the cellulose was less ordered in the dried state. Although, in case of cellulose, loss of spectral resolution also arises upon reduction of its crystallinity (Agarwal et al., 2010), it was found that the cellulose crystallinity remained unchanged upon drying. Therefore, the lower spectral resolution reflected the conformational changes of cellulose chains in the sample. It is possible, however, that the chains that exist on the outer surface of the fibrils are more strongly affected compared to the chains in the interior. Such changes in band positions, shapes, and intensities are likely to arise from changes in the H-bonds between neighboring cellulose molecules and/or between water and cellulose molecules.

### Low frequency region

**Figure 11:**
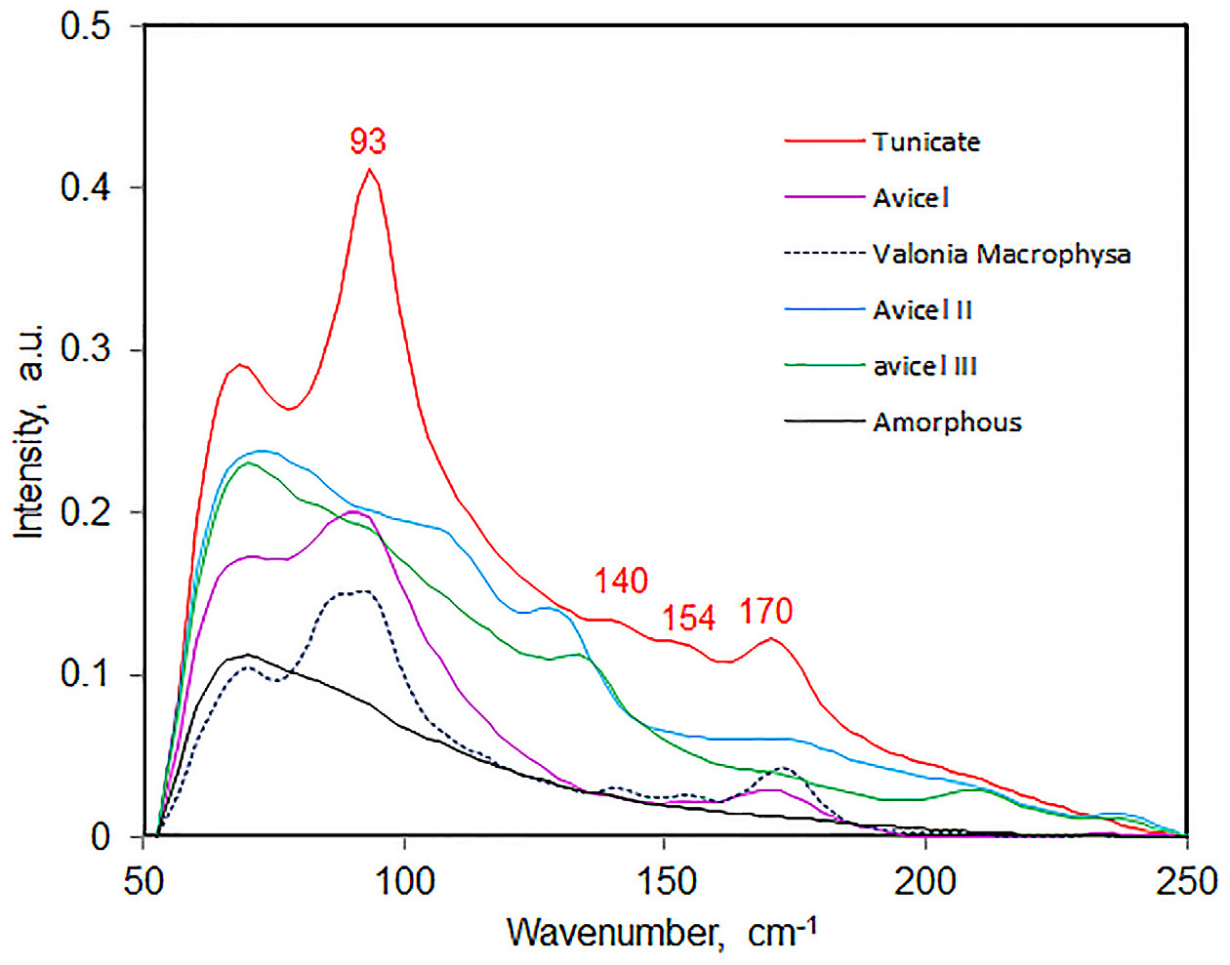
**Low frequency (50–250 cm^−1^) Raman spectra of various celluloses in dried state**.

Low frequency vibrational modes have been difficult to detect in conventional Raman spectroscopy because such bands exist over the intense wings of the Rayleigh-line. However, this situation was significantly improved with the availability of 1064-nm excitation based FT-Raman instruments where the lower frequency limit was found to be around 80 cm^−1^. It is possible, for the first time, to observe directly Raman scattering from such modes in celluloses. Considering that the H-bonds are low energy vibrations, in a Raman spectrum, these are likely to appear in the low frequency as well. In Figure 11, spectra in the 50–250 cm^−1^ region are reported for a number of cellulose materials—tunicin (cellulose I_β_), valonia macrophysa (cellulose I_α_), avicel, avicel II, avicel III_I_, and amorphous cellulose. Except for amorphous cellulose which is completely disordered bands were detected for all other celluloses (Table 4). In the 50–250 cm^−1^ region, primarily contributions from crystalline cellulose polymorphs are detected. In Figure 11, band positions are annotated only for tunicin cellulose while for others this information is provided in Table 4. Polymorphs of cellulose can be distinguished based on the information in the low frequency region. For instance, cellulose I_α_ and cellulose I_β_ can be distinguished due to the presence of an extra strong feature at 87 cm^−1^ in I_α_ which is absent in I_β_. Similarly, cellulose II has the contributions at 106 cm^−1^ and 128 which are not detected in other forms of celluloses (Table 4). In addition to the differences in band positions, band intensity and shape varies which can also be used for making distinctions between materials. Although, currently, bands associated with various H-bonds have not been assigned, as further progress is made, this region is likely to provide important new information on existence of different kinds of H-bonds and on formation and breaking of such bonds. The information from low frequency Raman is likely to be important for research in field of biomass materials.

**Table 4:** **Raman peak positions in the low frequency region, cm^−1^**.

**Tunicate (I_β_)**	**Valonia macrophysa (I_α_)**	**Avicel (I)**	**Avicel (II)**	**Avicel (III_I_)**	**Amorphous**
–	–	–	210 (m)	210 (m)	–
174 (m^*^)	172 (m)	170 (w-m)	173 (sh)	170 (vw)	–
154 (w)	153 (w)	155 (vw)	–	–	–
140 (w)	139 (w)	140 (vw)	–	134 (m)	–
–	–	–	128 (sh)	–	–
–	–	–	106 (sh)	–	–
93 (s, br)	92 (s, br)	91 (m, br)	–	93 (vw)	–
–	87 (s, br)	–	81 (vw)	83 (vw)	–

* *Relative band intensity; s, strong; m, medium; w, weak; vw, very weak; br, broad; sh, shoulder*.

### Implications

Raman spectroscopy is capable of analyzing chemical bonds at the molecular level. What that means is that it is capable of detecting molecular species in structurally complex systems such as plant tissues and plant derived materials wherein many molecules coexist and have complex intra- and inter-molecular associations. Given the capability of generating the kind information described here, the areas of research where Raman spectroscopy can contribute productively are unlimited. Basically, it comes down to the nature of questions being asked in these fields. Such fields vary from old, such as wood and paper, to new, like cellulose nanocomposites and cellulose ethanol. Then there are implications for advancing basic understandings in the areas of plant science where how inhomogeneous, anisotropic, and hierarchical structures support tissue functions need better understanding. Role of 1064-nm Raman spectroscopy is illustrated below by a couple of examples. In the context of enzymatic conversion of biomass to ethanol, an area of producing bioenergy, the role of cellulose crystallinity has been controversial (Agarwal and Ralph, 2014, and references cited therein). More specifically, the question is, is the cell wall crystallinity responsible for the recalcitrant behavior of the biomass? The findings, partly based on critical information from Raman spectroscopy, indicated that intrinsic crystallinity of woody tissue was not detrimental to the enzyme hydrolysis. Similarly, the Raman capability seems to be perfectly suited for the detailed analysis of cellulose structure which is intimately associated with other molecules in plant cell walls. Some novel information was recently obtained which suggested that plant-cellulose at the microfibril level may not be crystalline at all (Agarwal et al., 2014). Analysis of cellulose structure is important in untangling its role in the cell wall biosynthesis and function in the cell wall. The mechanism of cell wall growth is considered to be controlled by cellulose structure and interactions between cell wall polymers while cellulose is being synthesized (Cosgrove, 2005). Understanding cellulose and other components in native state will advance our knowledge of cell wall which in turn will provide understanding of areas of plant structure and physiology—areas such as how cell wall toughens the plants, how plant grows, and how cell differentiation occurs.

## Conclusions

Investigations of plants and plant materials carried out in our laboratory with 1064 nm FT-Raman spectroscopy were considered: these studies resulted in general characterizations of the materials, comparisons of compression wood with normal wood, assessment of cellulose crystallinity, and quantitation of lignin content in walls of secondary tissues. Of particular importance, cellulose was compared in the hydrated and dried states. Conformations of cellulose molecules and the interaction of water with cellulose were of particular interest. The study of cell walls has suffered from lack of physical techniques to elucidate the biophysical complexities of their compositions and structures. Whereas modern methodologies have rapidly expanded our understanding of plant cytoplasm, understanding of the cell wall has lagged for lack of sufficient biophysical tools. In this regard, the capabilities of FT-Raman methodology are generally underappreciated and underutilized. They are expected to play important roles in developing a fuller appreciation of the wall as a critical feature of the plant and an essential area for application of biotechnology.

### Conflict of interest statement

The author declares that the research was conducted in the absence of any commercial or financial relationships that could be construed as a potential conflict of interest.
